# Novel exosome-related risk signature as prognostic biomarkers in glioblastoma

**DOI:** 10.3389/fimmu.2023.1071023

**Published:** 2023-02-14

**Authors:** Mingyan Ding, Qiang Xu, Xiuying Jin, Zhezhu Han, Hao Jiang, Honghua Sun, Yongmin Jin, Zhengri Piao, Songnan Zhang

**Affiliations:** ^1^ Department of Oncology, Yanbian University Hospital, Yanji, China; ^2^ Department of Radiation Oncology, Yanbian University Hospital, Yanji, China

**Keywords:** exosome, risk score, glioblastoma, machine algorithm, tumor microenvironment, immunotherapy

## Abstract

Exosomes are progressively being detected as an indicator for the diagnosis and prognosis of cancer in clinical settings. Many clinical trials have confirmed the impact of exosomes on tumor growth, particularly in anti-tumor immunity and immunosuppression of exosomes. Therefore, we developed a risk score based on genes found in glioblastoma-derived exosomes. In this study, we used the TCGA dataset as the training queue and GSE13041, GSE43378, GSE4412, and CGGA datasets as the external validation queue. Based on machine algorithms and bioinformatics methods, an exosome-generalized risk score was established. We found that the risk score could independently predict the prognosis of patients with glioma, and there were significant differences in the outcomes of patients in the high- and low-risk groups. Univariate and multivariate analyses showed that risk score is a valid predictive biomarker for gliomas. Two immunotherapy datasets, IMvigor210 and GSE78220, were obtained from previous studies. A high-risk score showed a significant association with multiple immunomodulators that could act on cancer immune evasion. The exosome-related risk score could predict the effectiveness of anti-PD-1 immunotherapy. Moreover, we compared the sensitivity of patients with high- and low-risk scores to various anti-cancer drugs and found that patients with high-risk scores had better responses to a variety of anti-cancer drugs. The risk-scoring model established in this study provides a useful tool to predict the total survival time of patients with glioma and guide immunotherapy.

## Introduction

Exosomes are disk-shaped vesicles with a diameter of 30-150 nm, containing complex RNA and a variety of proteins, recognized as important regulators in the early stages of cancer genesis and progression, according to mounting data (1). Research on the anti-tumor immunity of exosomes can be traced back to the exploration of exosomes by Zitvogel et al (2).at the end of the 20th century. A large number of clinical trials have verified the impact of exosomes on tumor growth, particularly in anti-tumor immunity and immune function inhibition. Exosomes carrying tumor antigens can use dendritic cells to improve anti-tumor immunity because such exosomes can present MHC antigen peptide complexes to dendritic cells. Tumor cell-derived exosomes express FasL, which increases the rate of apoptosis and inhibits the differentiation of dendritic cells (3).

Glioblastoma (GBM) is a tumor derived from the neuroepithelium. Statistics show that gliomas account for more than 30% of primary brain tumors and are more common among intracranial malignant tumors (4). Gliomas can also be classified as glioblastoma multiforme, astrocytoma, and medulloblastoma (5). Infiltrative growth is the main feature of gliomas. Gliomas invade multiple brain lobes and destroy brain tissues during their growth. In general, traditional tumor treatment methods are ineffective in treating patients with GBM. The median overall survival (OS) of patients with GBM after chemoradiotherapy was 14.4 months. In the treatment of numerous malignancies, immune checkpoint inhibitors like PD-1/L1 and CTLA-4 have displayed astounding clinical efficacy (6). A small percentage of glioma patients, however, respond well to the current checkpoint treatment. It is consequently critical to creating more effective immunotherapies for gliomas.

The immune system is the most important line of defense of the body against tumor attacks. Tumor cells perform tumor immune escape by abnormally expressing related miRNAs (7). Exosomes play a bridging role in the process of information transmission, induce apoptosis of natural killer cells, inhibit the differentiation of dendritic cells, and promote immune escape. miR-130b and miR-32 in exosomes can stimulate the potential of the body’s autoimmune system, regulate phosphatase (PTEN) deletion on human chromosome 10, improve the transformation speed of M2 macrophages by PI3K/Akt signal transduction, and create a shortcut for glioma growth and migration (8).Several clinical trials have confirmed that hypoxic glioma-derived exosomes can promote glioma proliferation and migration (9). It uses miR-1246 to target the telomere repeat binding factor 2 binding protein (terf2ip), successfully activating the signal transducer and activator of transcription 3 (STAT3) signaling pathway and simultaneously inhibiting the nuclear transcription factor kappa B (NF-κB) signaling pathway (10). During the formation and growth of glioma, miR-21 in glioma-derived exosomes can directly downregulate targets such as BTG 2, PDCD 4 and NFAT5, thereby expanding the infiltration range and increasing the proliferation rate of microglia, the innate immune cells of the nervous system (11). Considering this, exosomes may represent a feasible target to alter the glioma patient’s tumor-associated immune milieu and make them more susceptible to immunotherapy.

In this study, we used the NMF clustering method to divide 273 exosome key genes into two groups and built a risk score using the genes screened by LASSO. We evaluated the relationship between the risk score and cellular components or cellular immune responses and compared the differences in immune responses under different algorithms. This in-depth research emphasizes the crucial part that tumor-derived exosomes play in determining the tumor-associated milieu and highlights their potential as a target for glioma immunotherapy optimization.

## Materials and methods

### Data collection and preprocessing

The Cancer Genome Atlas (TCGA) datasets, UCSC Xena (https://xenabrowser.net/), were used to retrieve the glioma gene-expression datasets and clinical annotations. The exosome-related gene expression of GSE106804 and three external validation datasets GSE 4412, GSE13041, and GSE43378 were downloaded from the Gene-Expression Omnibus (GEO; https://www.ncbi.nlm.nih.gov/gds/). The downloaded RNA sequencing (RNA-seq) data converted the number of fragments per million fragments (FPKM) into transcripts per million terabytes (TPM). Microarray data were generated using Affymetrix and Agilent, and quantile normalization and background correction were performed using the RMA algorithm.All data in the analysis process were analyzed using R software (version 3.6.1) and the R Bioconductor software package.

### Identification and clustering of the exosome pattern genes

The exosome pattern genes were identified by two cohorts between 13 GBM and 6 normal samples in GSE106804. The R package limma was performed by significance criteria (P.Value< 0.05 & logFC> 1) (12). Using the NMF clustering method, the exosome pattern genes were classified into two clusters (13).

### Establishment of the exosome-related risk signature

Univariate Cox regression analysis and Random Survival Forest were used to reduce the dimensionality of genes (14). Tuning parameter selection was performed by 1000 rounds of cross-validation to prevent overfitting and the partial likelihood deviance met the minimum requirements. A set of prognostic genes and their LASSO regression coefficients were obtained. The selected lasso gene was used to construct a risk score whose expression value was the sum of the LASSO regression coefficients (**Supplementary Table 1**).


0.2829*IGFBP6 + 0.1751*VGF + 0.2063*TRBC1

The time-dependent receiver-operating characteristic (ROC) and Kaplan-Meier(K-M) curves were used to assess the clinical prognostic capacity of the exosome-related risk score using the R “survival”, and “survminer” packages.

### Estimation of immune characteristics and immune infiltration

To evaluate the relationship between exosome-related risk score and cellular component or cellular immune response, the CIBERSORT (15), ESTIMATE (16),McCounter (17),ssGSEA (18),and TIMER Algorithms (19)were compared. A Heatmap was used to uncover differences in immune response under different algorithms.

### Functional and pathway enrichment analysis

All gene sets were downloaded from the MSigDB database. Gene set enrichment analysis (GSEA) were performed using the clusterProfiler R package. Pathways enriched in exosome pattern genes were identified in Gene Ontology (GO) and Kyoto Encyclopedia of Genes and Genomes (KEGG) with a strict cutoff of P<0.05.

### Prediction of immunotherapy response

A cohort of urothelial carcinomas treated with atelizumab is used to predict immunotherapy response in the IMvigor210 trial (http://research-pub.gene.com/IMvigor210CoreBiologies/) (20). A dataset (GSE78220) used to predict PD-1 immunotherapy response (pembrolizumab or nivolumab) was also utilized (21). We also explored the immune and genomic correlates of response to anti-PD-1 immunotherapy in glioblastoma.With a license from Creative Commons 3.0, complete expression data can now be downloaded from research-pub.gene.com/imvigor210corebiologies, and complete clinical data can be retrieved and downloaded. The raw data were standardized using the deseq2r software package, which converts the counts to TPM values and the FPKM-normalized values to TPM values. This creates a separate risk score for both datasets.

### Statistical analysis

The Shapiro–Wilk normality test was used to test the normality of the variables. For normally distributed variables, unpaired Student’s t-tests were used to compare differences between the two groups. The Wilcoxon test for variables was used to compare non-normal distributions. Pearson’s correlation and range correlation were used to calculate the correlation coefficients. Data were visualized using the R package ggplot2. The random survival forest package was used to create a random survival forest. The survival ROC curves were plotted using the timeROC package, and the survival curves of the subgroups were generated and visualized using the Kaplan–Meier method. All survival curves were generated using the R packet survminer. All the heat maps were based on a pheatmap. All statistical analyses were performed using R software (https://www.r-project.org/, edition 3.6.1). All tests were two-sided, and P values<0.05 were considered statistically significant. Waterfall diagrams were implemented using the maftools package, and both GSEA and enrichment analyses were implemented using the R package cluster profiler.

## Results

### Identification of exosome pattern genes in GBM

**Figure 1:**
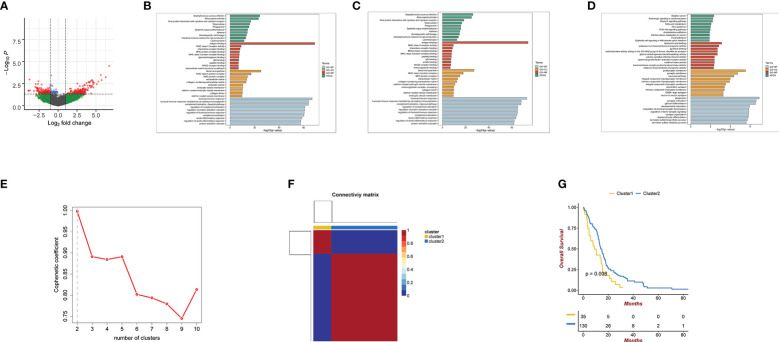
**(A)** Volcano map of cluster1 and cluster2 differential genes. Green coloration denotes genes with FC cutoff but no P cutoff. Green represents genes that meet FC cutoff standards but fail to meet P cutoff standards. Blue represents genes that meet FC cutoff standards but fail to meet P cutoff standards.Red represents genes that both meet FC cutoff and P cutoff standards.Gray represents genes that both meet FC cutoff and P cutoff standards **(B)** GO and KEGG analyses for Exosome pattern genes. **(C)** GO and KEGG analyses for cluster1. **(D)** GO and KEGG analyses for cluster2. **(E, F)** using NMF clustering method, the best clustering of exosome pattern genes is cluster1 and cluster2. The phenotypic coefficient decreases with the number of clusters. **(G)** Kaplan-Meier curves for the cluster1 and cluster2 differential genes. P<0.05.

A total of 273 exosome pattern genes were preliminarily screened by limma(P.Value<0.05 & logFC> 1) (**Supplementary Table 2**).The exosome pattern genes were divided into two groups using the NMF clustering method: cluster1 and cluster2. The comprehensive effectiveness increased with an increase in the number of clusters (**Figures 1E, F**). Survival analysis of cluster1 and cluster2 showed that there were significant differences between the two groups (**Figure 1G**). **Figure 1A** shows a volcano map of the two differential genes. These results indicate that there are significant differences between the cluster1 and cluster2 groups. The GO, KEGG analysis of exosome pattern genes were performed: BP participated in humoral immune response, humoral immune response mediated by circulating immunoglobulin, regulation of protein activation cascade, and acute inflammatory response; MF mainly regulated antigen binding, MHC class II receptor activity, and chemokine receptor binding, among others were mainly upregulated in blood microparticles, MHC class II protein complex, and MHC protein complex synthesis pathways. KEGG analysis revealed that the exosome pattern genes were mainly involved in Staphylococcus aureus infection, rheumatoid arthritis, viral protein interaction with cytokines and cytokine receptors, tuberculosis, phagosomes, systemic lupus erythematosus, asthma, hematopoietic cell lineage, intestinal immune network for IgA production, and leishmaniasis (**Figure 1B**).The GO, KEGG analysis of cluster 1 was performed: The immune response of BP was similar to that of exosome pattern genes.MF mainly regulated antigen binding. KEGG analysis revealed that Cluster 1 was mainly involved in Staphylococcus aureus infection and rheumatoid arthritis (**Figure 1C**).The GO, KEGG analysis of cluster 2 was performed: BP participated in gliogenesis,synapse maturation,glial cell differentitation and so on. MF mainly regulated hyaluronic acid binding. KEGG analysis revealed that Cluster 2 was mainly involved in gliogenesis, synapse maturation,glial cell differentitation and so on (**Figure 1D**).

### Generation of prognostic gene signature and its functional annotation

**Figure 2:**
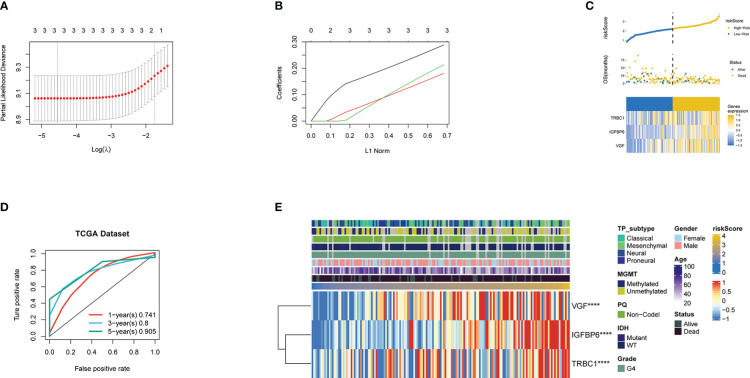
**(A)** Confidence interval under each lambda. **(B)** Trajectory of each independent variable, wherein the horizontal axis represents the log value of the independent variable lambda and the vertical axis represents the coefficient of the independent variable. Three lines of different part colors represent the three prognostic genes. **(C)** Risk Score, survival status and the expression of three genes in the whole TCGA dataset. **(D)** ROC curve and AUC of the three-gene signature.ROC curve measuring the sensitivity of risk score in predicting the 1-year, 3-year, and 5-year survival of the patients. The area under the ROC curve was 0.741, 0.8, and 0.905, respectively. **(E)** Heatmap of the three prognostic genes. **** Represents P <0.0001.

**Figure 3:**
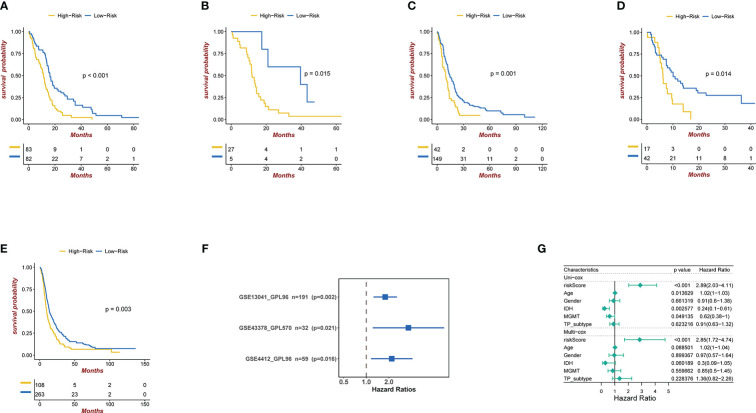
**(A)** Kaplan-Meier curves for the TCGA patient groups with high and low risk scores. Test of log-rank, P< 0.001. **(B-D)** Kaplan-Meier curves in GSE4412, GSE13041, and GSE43378 for patient groups with high and low risk scores. Test of log-rank, P<0.001. **(E)** Kaplan-Meier curves for the CGGA patient groups with high and low risk scores. Test of log-rank, P< 0.05. **(F)** Univariate cox regression analyses analysis to assess the clinical prognostic value of independent glioma datasets in low/high risk groups. The hazard-ratio scale is log2 scaled. **(G)** Univariate and multivariate analysis regression analysis evaluates the clinical prognostic value of independent glioma datasets in low/high risk groups.

Univariate analysis was used to screen 28 genes related to prognosis, and the random survival forest algorithm was used to rank the importance of prognosis-related genes. Lasso regression analysis was used to further reduce and validation the number of genes, and finally, three prognostic genes were screened, and a risk-scoring model was constructed (**Figures 2A, B**). To demonstrate the signature’s capacity to be distinguished, the risk score splits the high-risk and low-risk categories based on the cutoff value (**Figure 2C**). Next, we decide to measure the capability of the model we developed using the Kaplan-Meier (K-M) method. The training cohort’s high-risk group has a lower chance of surviving than the low-risk group, as shown in **Figure 3A**,which is statistically significantly different (p<0.001). The ROC analysis indicated that area under the curve (AUC) for three-gene signature risk score reached 0.905 (**Figure 2D**). **Figure 2E** shows that the three signature genes are correlated with the risk score. GSEA was used to analyze the regulatory relationship between riskScore and pathways. GO functional enrichment analysis found that the risk Score positive regulation dephosphorylation, immune response regulating cell surface receptor signaling pathway among others. KEGG pathways enrichment analysis found that the risk Score positive regulation calcium signaling pathway, chemokine signaling pathway, and jak stat signaling pathway among others. (**Supplementary Figures 1**, **2**) (**Supplementary Tables 3**, **4**).

### Confirmation of the prognostic capacity of the three exosome related genes signature

We applied our prognostic classifier to the external validation sets GSE13041, GSE43378, GSE4412 and CGGA in order to validate whether it had similar predictive abilities in various populations. As shown in **Figures 3B–E**, the clinical prognostic value of the high-risk and low-risk groups in the four independent glioma datasets was different, and Survival analysis showed that patients with high-risk scores had worse prognoses than those with low-risk scores. The univariate and multivariate Cox regression analyses showed that exosome related risk score is the independent prognostic factors (p<0.01) (**Figures 3F, G**).

### Correlation between the tumor immune microenvironment and the risk score

**Figure 4:**
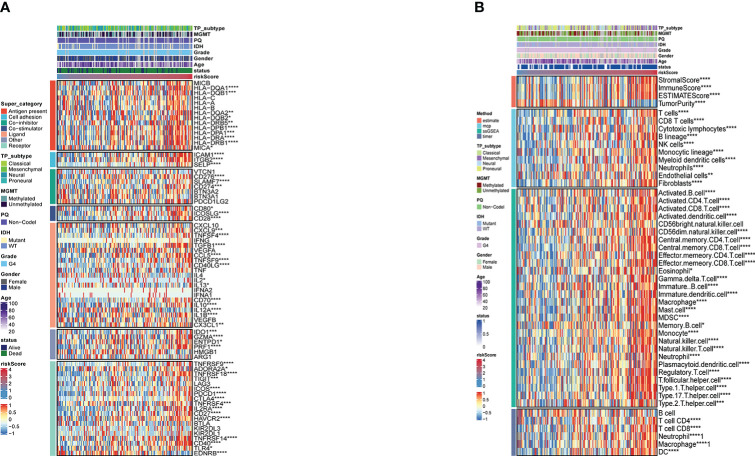
**(A)** Risk score-based immune checkpoint expression heatmap **(B)**. Heatmap for immune responses based onESTIMATE, McCounter, ssGSEA and TIMER algorithms in risk store. *P <0.05; **P <0.01; ***P <0.001; ****P <0.0001.

A comparison was first made between the risk score and immune modulators (22), which were divided into seven categories: antigen-presenting molecules, co-stimulator molecules, co-inhibitors, cell adhesion proteins and receptors, ligands, and others. Most immune checkpoint molecules were positively correlated with an increased risk score (**Figure 4A**). Thereafter, we analyzed the correlation between prognosis and immune infiltration. **Figure 4B** shows the relationship between the risk score and cell composition or cellular immune response using the ESTIMATE, McCounter, ssGSEA, and TIMER algorithms. Heatmaps revealed differences in the immune response under different algorithms. The McCounter algorithm indicated that higher risk score is associated with more immune cell infiltration. Including T cells, CD8 T cells, Cytotoxic lymphocytes, B lineage, NK cells, Monocytic lineage, Myeloid dendritic cells, Neutrophils, and Fibroblasts. According to the ssGSEA methodology, more immune cell infiltration is correlated with higher risk scores. The cells involved are shown in **Figure 4B**. Simply put, a higher risk score meant that there had been greater immune cell infiltration. In accordance with the current CNS tumor classification recommendations, we conducted a survival analysis of glioblastoma IDH wild-type.As shown in **Supplementary Figure 4**, the statistically significant difference (p<0.001) between the survival rates of the TCGA samples from the high-risk idh-wt group and those from the low-risk group.

### Genomic features of the exosome-related gene pair score groups in glioma

**Figure 5:**
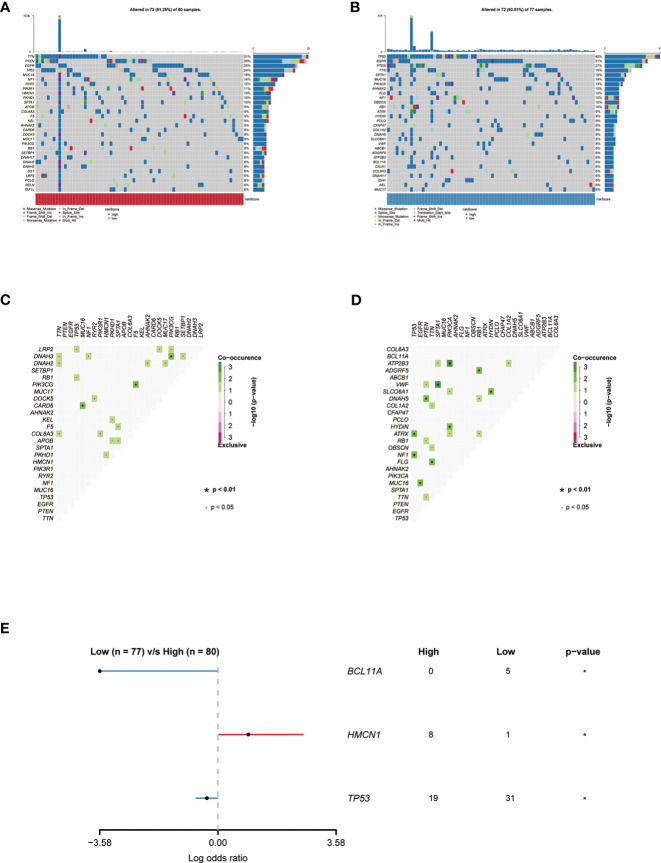
**(A)** Genes with the highest frequency of somatic mutations in the group with high risk scores are listed below. **(B)** Genes with the highest frequency of somatic mutations in the group with low risk scores are listed below. **(C, D)** The heatmap showing the concurrence or mutual exclusivity of the top 25 most mutated genes in the two clusters.p< 0.05, *p< 0.01. **(E)** The Forest plot listing the top 3 most mutated genes between the two clusters.

Mutation landscapes in the high- and low-risk groups were compared, and somatic mutation analysis revealed that more mutation events occurred in samples with lower risk score group.TP53(40%) and EGFR (31%) mutations were more prevalent in the low-risk score group. In the high-risk group, TP53(24%) and EGFR (25%) mutations were significantly different from those in the low-risk group (**Figures 5A, B**). Except for NF1 and RB1, which frequently had frame-shifting deletions, missense mutations were the most common kind of gene modification in all of these genes. The strongest co-occurrent pairs of gene alteration in high-risk score group were CARD6-TP53 and PIK3CG-F5, DNAH3-PIK3CG (**Figure 5C**).VWF-SPTA1 and ATP2B3-PIK3CA were the strongest co-occurring gene pair changes in the low-risk score group. (**Figure 5D**). **Figure 5E** lists the top 3 most frequently altered cancer-related genes.

### Comparison of anti-cancer drug sensitivity based on exosome-related risk score

We compared sensitivity to 56 anti-cancer drugs in patients in the high and low risk score group to identify potential treatment modalities. The result shows that the IC50s of Axitinib, ARTA, AKT, BIRB.0796, CCT007093, Cisplatin, Cyclopamine, and Doxorubicin were significantly higher in patients with high-risk score group. The IC50s of Bicalutamide, BMS.754807, Bosutinib, and Bryostatin.1 were significantly higher in low-risk score group (**Supplementary Figure 3**).This effectively demonstrates the significance of the exosome-related gene signature.

### The inference of the benefaction of risk signature in immunotherapy

**Figure 6:**
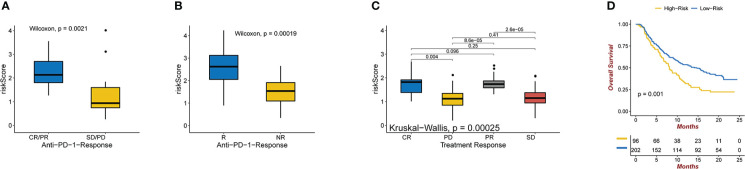
**(A)** Anti-PD-1 clinical response status groups (CR/PR and SD/PD) with varying risk scores. Wilcoxon test was used to compare group differences (P = 0.0021 for Wilcoxon). **(B)** Immune and genomic correlates of response to anti-PD-1 immunotherapy in glioblastoma(P = 0.00019 for Wilcoxon). **(C)** Various anti-PD-1 clinical response statuses in groups with varying risk scores (CR, PR, SD, PD). The Kruskal-Wallis test was used to compare group differences (Kruskal-Wallis, P = 0.00025). **(D)** In the IMvigor210 dataset, Kaplan-Meier curves were created for the two risk score groups. Test of log-rank, P = 0.001.

By dividing up the patients in the melanoma dataset (GSE78220) cohort into various risk risk score groups, the capacity of the risk score to predict the response of patients to immune-checkpoint therapy was investigated. High-risk patients showed improved immunotherapeutic outcomes (**Figure 6C**).The ability of the risk score to predict a patient’s response to immune-checkpoint therapy was examined by segmenting the patients in the GBM dataset (SRP155030) cohort into different risk score groups.Patients at high risk had better responses to immunotherapy (**Figure 6B**) (23). The capacity of the risk score to predict the response of patients to immune checkpoint therapy was explored by relegating the IMvigor210 cohort patients (urothelial carcinoma dataset) to various risk score groups. Patients with a high-risk score exhibited better immunotherapeutic responses (**Figure 6A**). Patients who received atezolizumab as the anti-PDL1 medication and had a high-risk score showed a significantly reduced overall survival (OS) compared to those who had a low-risk score. (**Figure 6D**).

## Discussion

Exosomes, which play a role in intercellular communication, are extracellular vesicles released by the majority of eukaryotic cells (24). Exosomes are important regulators of cancer initiation and progression, and growing data show that they can promote the malignancy of gliomas by inhibiting the immune system or changing the tumor microenvironment (25–27).As exosomes are durable in peripheral blood, they are prospective tumor-derived materials for the characterization of tumor behavior. Exosomes can be monitored, and exosome-derived proteins and RNAs can also be used for diagnosis (28). As a result, it appears that analyzing the molecular make-up of exosomes released by glioblastoma cells is a very promising avenue for the creation of non-invasive diagnostic techniques for this disease (29).

To explore and confirm the predictive value of the exome in glioma, we developed a risk score based on genes found in tumor-derived exosomes. The risk score provided information on immunological and stromal states and predicted survival in glioma patients. Between low- and high-risk scores, there were notable discrepancies, showing high efficiency in predicting patients 3 years and 5 years survival probability. Cox regression analysis showed that the risk score was a better independent prognostic factor in patients with glioma than in those with other characteristics (P<0.001). Three novel tumor-derived exosome genes, insulin‐like growth factor binding protein 6 *(IGFBP6), VGF* (non-acronym), and T-cell Receptor Constant β Chain-1 (*TRBC1*), which significantly affected the risk score, were also identified. *IGFBP6* is a soluble binding protein that is a part of the insulin-like growth factor *(IGF*) system (30). Through paracrine IGF2/IGF-1R signaling, *IGFBP6* regulates the growth of chemoresistant glioblastoma, which is produced by scilicet chemosensitive tumor cells, and is secreted, which slows the evolution of GBM (31). The IGF-I receptor (*IGF-IR*) may be a promising target for GBM therapy because prior research has shown that GBMs overexpress *IGF-IR* and insulin-like growth factor receptor II (*IGF-IIR*) relative to the normal brain (32). One neuropeptide, *VGF*, has been linked to several mechanisms of action (33).Pro-BDNF is converted into mature the Brain Derived Neurotrophic Factor (BDNF) by the action of *VGF*, which also phosphorylates its receptor tropomycin receptor kinase B (TrkB) in an auto-regulatory loop induced by BDNF (34). An earlier study showed that immature dentate granule cells (DGCs) emit brain-derived neurotrophic factor (BDNF) and that glioblastoma stem cells (GSCs) express neurotrophic receptor kinase 2 (NTRK2), also known as TrkB, an appropriate receptor for BDNF (35). According to a study, BDNF-NTRK2 signaling promotes the AKT pathway to support GSC survival and development (36). By inhibiting the BDNF-NTRK-AKT-VGF axis, it may be possible to stop the DGC-GSC connection from generating tumors. *TRBC1* is a T cell receptor-chain constant region with significant immunotherapeutic potential for T cell malignancies (37).

In recent years, increasing studies have demonstrated that the tumor immune microenvironment is crucial for the emergence of cancer (38). The use of immunotherapy for cancer treatment is a novel concept (39). There remains a huge challenge in identifying a novel method for classifying patients who would benefit from immunotherapy. Next, we attempted to create a solid link between the risk score and tumor immune microenvironment. The high-risk group also expressed more immune checkpoint markers, such as *ICAM-1, CCL-5, PDCD1*, and *CXCL9*, and had a higher ESTIMATE score, which tended to correspond to immune-invading cells. The effects of therapeutic inhibitors that block PD-1/PD-L1 on immunotherapy have been demonstrated in a previous study on multiple cancers, which is consistent with our results that the risk score could predict an effective treatment (40). To date, anti-PD-1 therapy has not been shown to confer survival benefits in patients with recurrent glioblastoma (41). Our study examined the effectiveness of anti-PD-1 therapy in the melanoma cohort GSE78220 based on the risk score. IMvigor210, a cohort of patients treated with atezolizumab in response to anti-PD-L1 antibodies, was used to analyze the immunotherapy responses (42). We further used glioma data to test the effect of response to PD-L1. Among patients with high-risk scores, anti-PD-1/PD-L1therapy was more likely to be beneficial, demonstrating that gliomas and melanoma have different immune invasive microenvironments. Therefore, we propose that the risk score may function as a sensitive measure for anticipating the response of glioma patients to anti-PD-1/PD-L1 therapy. The risk score developed in this study may also allow doctors to choose different anti-cancer medications to treat gliomas. To identify prospective treatment methods, the sensitivity to several anti-tumor medications was evaluated between the high- and low-risk score groups in this study.

A comparison of exosomal genes and risk scores led to significant results. This study provides information that enables us to increase the number of potential exosomal biomarkers and glioma prognostic indicators. To thoroughly evaluate exosome biomarkers compared to routine clinical signs, substantial randomized control trials are required. Our research recommends the development of non-invasive methods for the diagnosis and prognosis of diseases using exosomes from brain tumors.This study was a retrospective bioinformatic analysis that was unable to perform senescence analysis and exhaustion phenotype analysis of immune cells infiltrating the tumor microenvironment at a single-cell level, which was a weakness of this study; In future prospective studies we will focus on this area of exploration.

## Data availability statement

The datasets presented in this study can be found in online repositories. The names of the repository/repositories and accession number(s) can be found in the article/**Supplementary Material**.

## Author contributions

SZ proposed the topic. MD, QX contributed to the design and interpretation of the study. MD, QX, XJ, ZH, HJ, HS, YJ, and ZP conducted the calculation and figures in R. All authors contributed to the article and approved the submitted version.
