# Supplementary material for: Novel exosome-related risk signature as prognostic biomarkers in glioblastoma
Source: Front Immunol. 2023 Feb 14;14:1071023. doi: 10.3389/fimmu.2023.1071023 (PMC9971586; doi:10.3389/fimmu.2023.1071023)

GO POSITIVE REGULATION OF DEFENSE RESPONSE

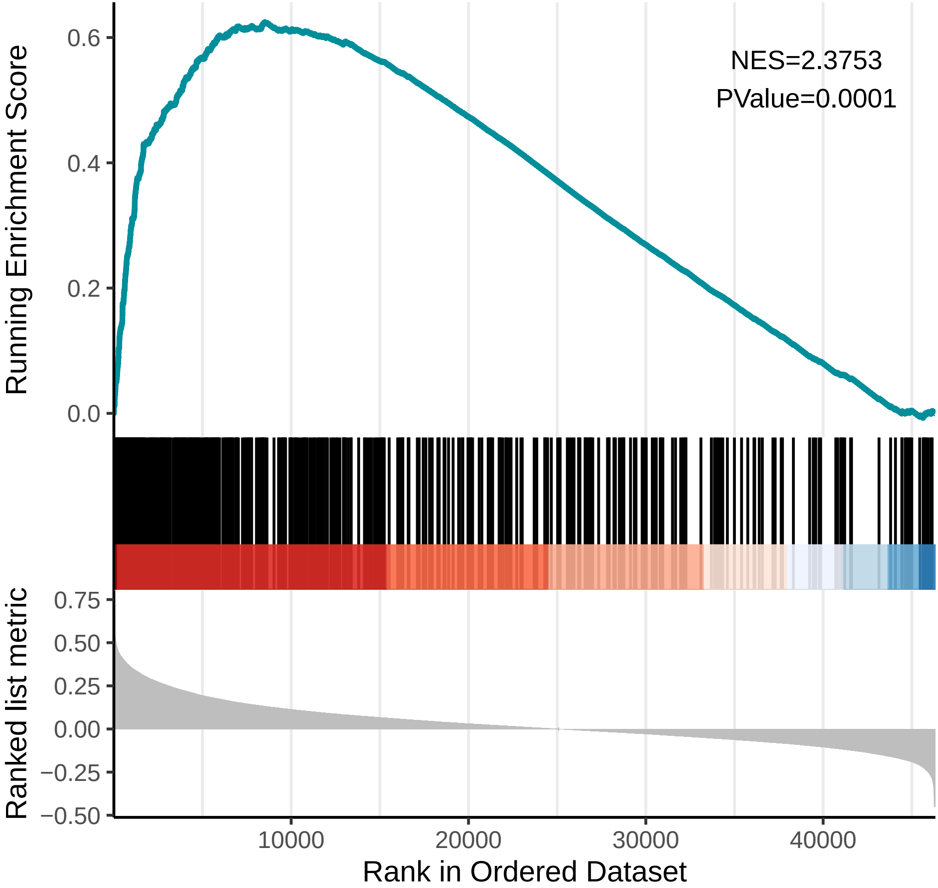

Supplement: Supplementary file 1 [file DataSheet_1.zip › 123/Supplementary Figure 1.zip!/GO_POSITIVE_REGULATION_OF_DEFENSE_RESPONSE.pdf]
