# Supplementary material for: Novel exosome-related risk signature as prognostic biomarkers in glioblastoma
Source: Front Immunol. 2023 Feb 14;14:1071023. doi: 10.3389/fimmu.2023.1071023 (PMC9971586; doi:10.3389/fimmu.2023.1071023)
Supplement: Supplementary file 1 [file DataSheet_1.zip › 123/Supplementary Figure 2.zip!/KEGG_T_CELL_RECEPTOR_SIGNALING_PATHWAY.pdf]

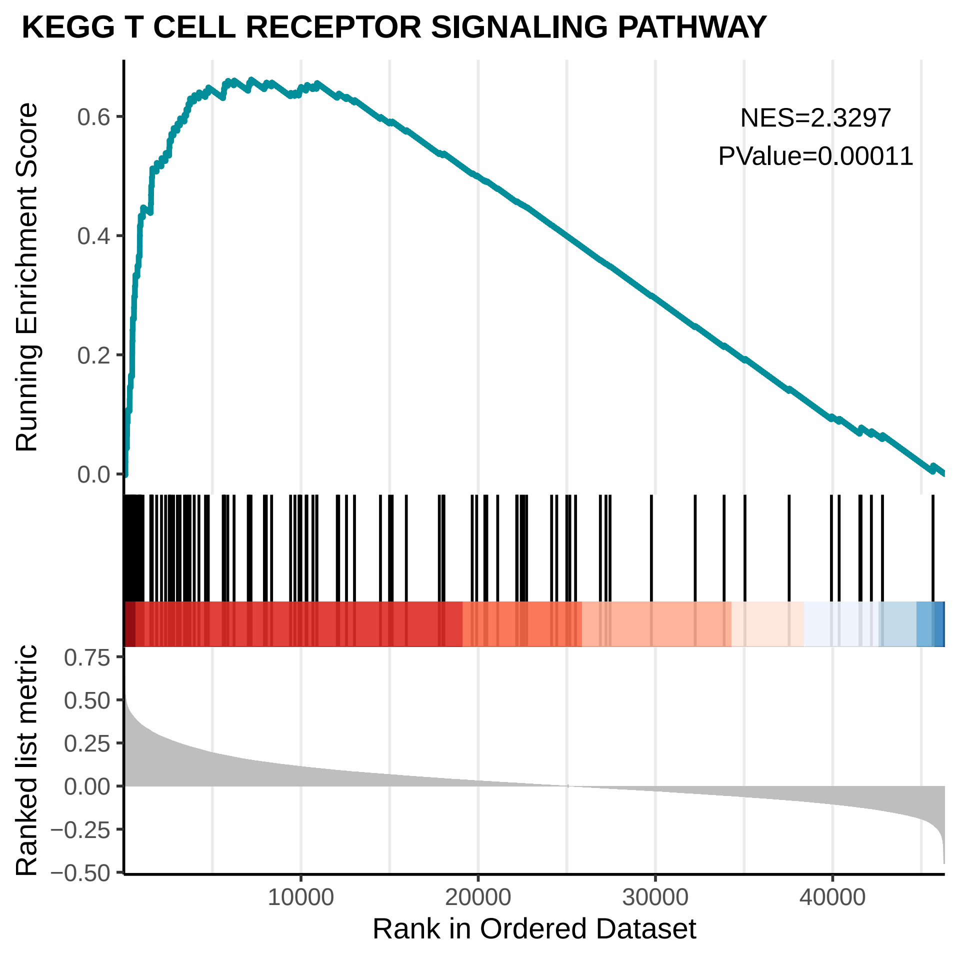
KEGG T CELL RECEPTOR SIGNALING PATHWAY
NES=2.3297
PValue=0.00011
Running Enrichment Score
0.6
0.4
0.2
0.0
Ranked list metric
0.75
0.50
0.25
0.00
−0.25
−0.50
10000
20000
30000
40000
Rank in Ordered Dataset
