# Supplementary material for: Novel exosome-related risk signature as prognostic biomarkers in glioblastoma
Source: Front Immunol. 2023 Feb 14;14:1071023. doi: 10.3389/fimmu.2023.1071023 (PMC9971586; doi:10.3389/fimmu.2023.1071023)
Supplement: Supplementary file 1 [file DataSheet_1.zip › 123/Supplementary Figure 3.zip!/Thapsigargin.pdf]

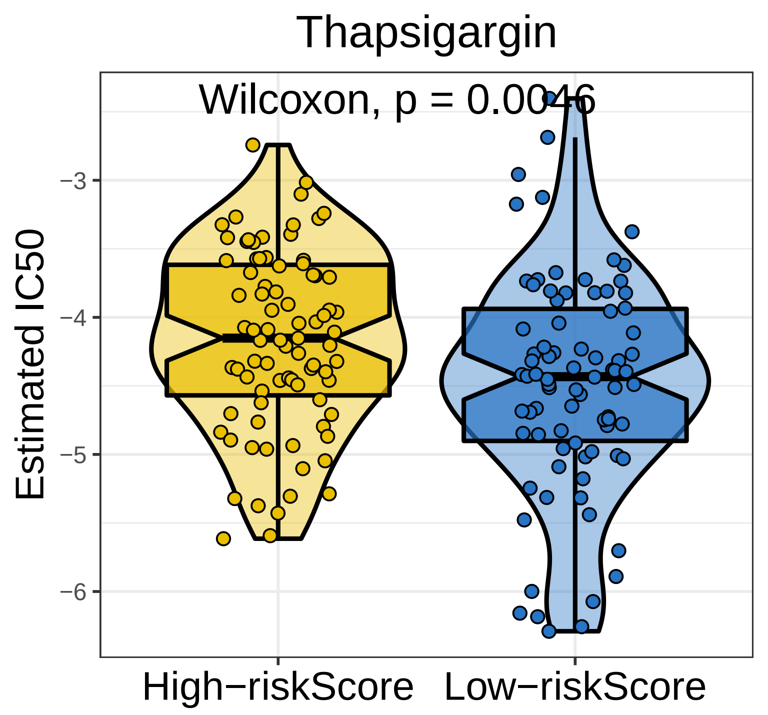

Thapsigargin
Wilcoxon, p = 0.0046
Estimated IC50
−3
−4
−5
−6
High−riskScore
Low−riskScore
