# Supplementary material for: Novel exosome-related risk signature as prognostic biomarkers in glioblastoma
Source: Front Immunol. 2023 Feb 14;14:1071023. doi: 10.3389/fimmu.2023.1071023 (PMC9971586; doi:10.3389/fimmu.2023.1071023)
Supplement: Supplementary file 1 [file DataSheet_1.zip › 123/Supplementary Figure 4.zip!/survplot_TCGA_lasso_IDH_WT.pdf]

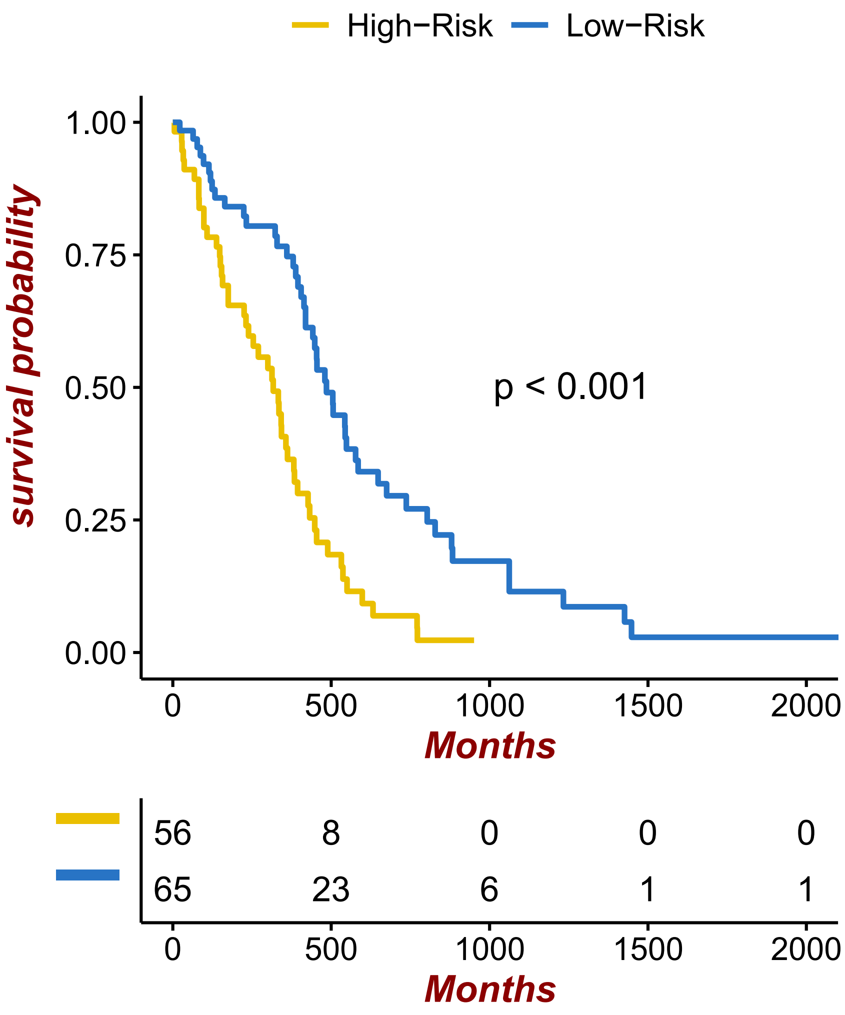

High−Risk
Low−Risk
survival probability
1.00
0.75
0.50
0.25
0.00
p < 0.001
0
500
1000
1500
2000
Months
56 8 0 0 0
65 23 6 1 1
0
500
1000
1500
2000
Months
